# AI – one size fits all? 

**DOI:** 10.5414/ALX02568E

**Published:** 2025-08-08

**Authors:** Stephan Traidl, Sonja Mathes, Sebastian Seurig

**Affiliations:** 1 Department of Dermatology and Allergy, Hannover Medical School, Hanover,; 2 Department of Dermatology and Allergy, School of Medicine and Health, Technical University of Munich, Munich, and; 3 Department of Respiratory Medicine, Allergology and Sleep Medicine, General Hospital Nuremberg, Campus North, Paracelsus Medical University, Nuremberg, Germany

**Keywords:** machine learning, artificial intelligence, AI, PCA, regression

## Abstract

The use of artificial intelligence (AI) in medicine requires a careful selection of suitable models, as there is no universal “one size fits all” method. While linear regression is convincing due to its simplicity and interpretability, it is limited due to the assumption of linearity and susceptibility to multicollinearity and outliers. More complex approaches such as neural networks show their strengths in the detection of non-linear patterns and automatic feature extraction but require large amounts of data, high computing capacity, and suffer from limited explainability. Principal component analysis (PCA) offers an efficient reduction of dimensionality. Ultimately, the choice of model depends on the balance between accuracy, interpretability, and data availability. A selection of machine learning models is presented in this article.

## Introduction 

Artificial intelligence (AI), is a collective term for the use of computers to perform tasks that typically require objective reasoning and understanding. To clarify terminology further: machine learning (ML) is considered a subset of AI and involves the use of statistical and mathematical modeling techniques that apply various approaches to automatically learn and improve the prediction of a target outcome without explicit programming [1]. Within the domain of ML, the term “deep learning” refers to the use of artificial neural networks for problem-solving. Due to the significance of ML and neural networks, John J. Hopfield and Geoffrey Hinton were awarded the Nobel Prize in Physics in 2024 for their contributions to this field [2]. However, the application of these technologies in medicine presents certain challenges, including biases, ethical considerations, data accessibility, model trust, and regulatory requirements [3]. ML is not an abstract concept but can be described as a multitude of different methods. This review article presents various ML techniques along with their advantages and disadvantages. Due to the breadth of the topic, only a selected set of methods is discussed to provide a general overview. 

### Supervised vs. unsupervised learning 

ML is fundamentally divided into supervised and unsupervised learning techniques [4]. There is also a method called reinforcement learning, which will not be discussed here. Supervised and unsupervised approaches pursue different goals and require specific data structures. Supervised learning is based on “labeled” data, meaning that the outcomes to be predicted (e.g., treatment response) are already known. It is frequently used in medical research to create prediction models, such as estimating the probability of treatment success. The main advantage of this approach is the high precision that can be achieved with well-structured and comprehensive datasets. However, it requires a large amount of labeled data, the generation of which can be time-consuming and costly. Moreover, imbalanced datasets can lead to biases and inaccurate predictions. 

In contrast, unsupervised learning does not require labeled data. This approach is particularly suitable for exploratory analyses, such as grouping patient cohorts with similar disease courses. 

**Figure 1:**
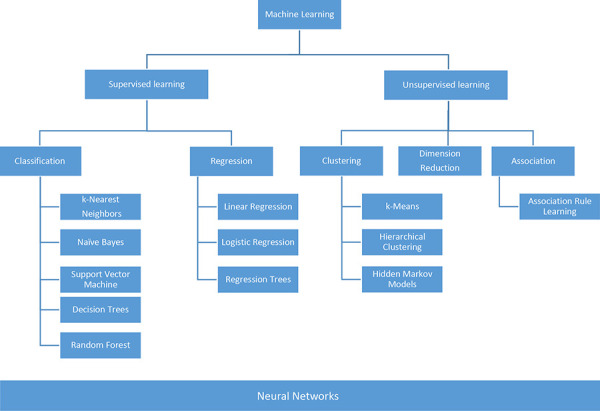
Schematic classification of various machine learning models. Division into supervised and unsupervised learning.

Figure 1 provides a conceptual classification of various ML methods. Within supervised learning, a distinction is made based on whether the outcome variable is categorical (e.g., yes/no) or metric (e.g., weight, height). The assignment of individual methods is not always clear-cut – for example, logistic regression – and varies between authors. 

### Linear regression 

Linear regression is one of the most fundamental models in ML. It describes linear relationships between independent and dependent variables and is primarily used in scenarios where the number of observations significantly exceeds the number of variables [5]. 

Its simplicity and ease of interpretability are its key strengths. It allows the direct quantification of the influence of individual variables and a clear representation of the underlying relationships between dependent and independent variables. This makes it a valuable tool in medical research and other fields where model transparency and interpretability are essential. Additionally, linear regression is widely applicable, especially when the underlying relationships are indeed linear or can be well approximated by linear models. 

However, a major limitation lies in the assumption of linearity, which may not adequately reflect many real-world scenarios. This significantly limits its applicability, as it cannot model non-linear relationships. Furthermore, linear regression is sensitive to outliers, which can distort results due to their disproportionate influence on coefficient estimation. Another issue is multicollinearity – the correlation between independent variables – which can affect the stability and interpretability of the model. Finally, underfitting may occur if the data’s complexity exceeds the model’s capacity, leading to poor fit and weak predictive performance. 

### Random forest 

The fandom forest approach is based on the combination of multiple decision trees whose results are aggregated into a final output. This method is suitable for both classification and regression problems [6]. 

**Figure 2:**
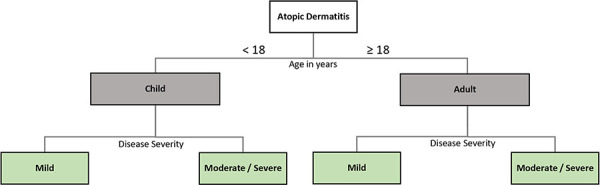
Example of a decision tree. Classification of atopic dermatitis by age and severity.

To understand the random forest method, it is necessary to first explain the decision tree. A decision tree is a graphical representation of decision processes organized in a tree structure. The data space is partitioned into several regions based on a series of “if-then” rules. Each decision represents a split, and the final leaves (leaf nodes) represent the outcome or classification. Figure 2 presents a decision tree designed to help classify patients with atopic dermatitis (AD). 

The core learning process involves identifying rules that best describe the data. This is done via an algorithm that optimizes the tree’s structure to make the best predictions. To determine the best decision at each node, the algorithm uses mathematical criteria such as information gain or Gini impurity. 

While an individual decision tree is easy to understand, it may suffer from overfitting if overly tailored to the training data. Random forests address this problem by combining many decision trees. They extend the decision tree concept by employing an ensemble of trees to generate more robust and accurate predictions. Each tree is trained on a random subset of the data and uses a random subset of features for decision-making. The individual results are then aggregated into an overall prediction. In the context of an AD prediction model, for example, the majority vote of trees might predict moderate to severe AD. 

A key advantage of random forests is their ability to model both linear and non-linear relationships in data. By combining multiple trees, the weaknesses of individual trees are mitigated, leading to more robust predictions. Moreover, random forests automatically select relevant variables and are less sensitive to outliers. These properties make them especially appealing in medicine, where data are often complex and heterogeneous. However, the method requires sufficiently large datasets, as it may be unstable with small sample sizes. 

### Artificial neural networks 

Neural networks are among the most advanced ML methods and are particularly effective for high-dimensional data such as images. They consist of multiple layers of neurons capable of capturing non-linear dependencies. For a detailed explanation, we refer to the article by Sebastian Seurig, “Machine Learning – what’s behind it” [9]. 

Compared to other methods, neural networks offer significant advantages, particularly their ability to recognize complex patterns in large datasets and model non-linear relationships. This makes them highly versatile for tasks such as image recognition, speech processing, and predictive modeling. They also feature automatic feature selection, extracting relevant characteristics from data without manual intervention. This is particularly valuable for problems where defining relevant variables in advance is difficult. 

Despite these advantages, neural networks also present major challenges. One key drawback is the lack of explainability and transparency. Because of their complex internal structure, they are often seen as “black-box” models. This poses a particular challenge in medicine, where decisions must be transparent and understandable. Approaches such as explainable AI (XAI) are promising but require additional effort and specialized expertise. 

Another concern is the potential amplification of biases present in training data. These biases can be inherited and even amplified by neural networks, leading to unfair or incorrect decisions – especially critical in sensitive areas like medical diagnostics. Furthermore, neural networks require large volumes of high-quality data. Without these, there is a greater risk of overfitting, where the model performs well on training data but poorly on new data. 

High computational requirements are another major drawback, as neural networks demand substantial resources for training and operation. This limits their use in resource-constrained environments. Finally, developing and implementing neural networks requires extensive technical knowledge, including expertise in ML and the specific data and application domain. 

### Principal component analysis 

Due to its widespread use in scientific literature, principal component analysis (PCA) will be discussed in more detail here [7, 8]. PCA is a dimensionality reduction technique used to reduce the number of variables in a dataset while preserving as much of the original information as possible. It identifies the principal axes (or components) of variation in the data, which are linear combinations of the original variables and represent the directions with the greatest data spread. 

**Figure 3:**
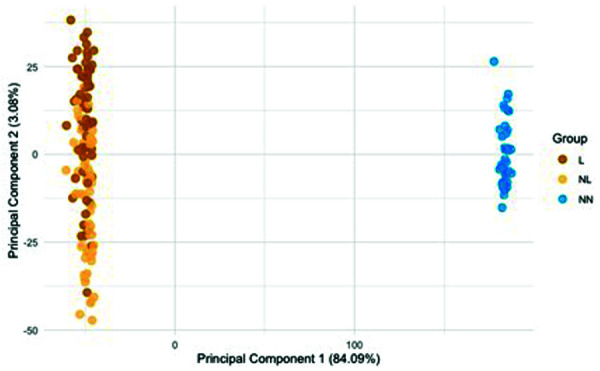
Example of a principal component analysis (PCA) based on transcriptome data from skin samples of patients with atopic dermatitis and healthy controls. L = lesional; NL = non-lesional; NN = healthy skin.

PCA offers several advantages, especially for reducing the dimensionality of high-dimensional datasets. By decreasing the number of variables to process, model training time can be significantly reduced, increasing efficiency. PCA also facilitates the visualization of such data, allowing projection onto two or three principal components to reveal patterns and trends that might not be visible in the original variables. Figure 3 illustrates an example PCA based on transcriptome data from skin samples of AD patients (lesional and non-lesional) and healthy individuals (NN). It shows a distinct grouping of healthy skin samples. Among AD samples, lesional (top, brown) and non-lesional (bottom, yellow) samples differ visibly. The variance explained by each principal component is usually reported (e.g., 84.09% by PC1). Another benefit is noise reduction, as less relevant or highly correlated variables can be eliminated, potentially improving model prediction accuracy. 

Despite its advantages, PCA has notable limitations. A primary concern is the potential loss of information during dimensionality reduction, which can impair the analysis’s accuracy and relevance. PCA is also sensitive to outliers, which can distort the principal components and results. Careful data preprocessing can mitigate this. Another drawback is the interpretability of results: since principal components are linear combinations of the original variables, their meaning is not always immediately clear. Additionally, determining the optimal number of components is challenging and often subjective, though tools like the scree plot can aid this decision by visualizing the explained variance. 

## Conclusion 

The choice of an appropriate ML method depends on the specific research question and the available data. There is no universal “one size fits all” model. While simple models like linear regression are valued for their interpretability, more complex approaches such as neural networks and random forests offer greater flexibility and adaptability. However, these often come at the cost of increased computational demands and reduced interpretability. Following the principle of Occam’s razor – which favors the simplest sufficient explanation – one should always ask whether a complex model is truly necessary to achieve the desired accuracy. Ultimately, finding the right balance between model complexity, accuracy, and transparency is key to successfully applying AI in medicine. Among multiple possible models, the one that solves the problem most appropriately and simply should be preferred. 

## Authors’ contributions 

ST drafted the manuscript. SM and SS contributed to the conceptualization of the work and provided critical revisions to the manuscript. All authors reviewed and approved the final version. 

## Funding 

No funding. 

## Conflict of interest 

No thematically relevant conflicts of interest.
